# Integrate Small RNA and Degradome Sequencing to Reveal Drought Memory Response in Wheat (*Triticum aestivum* L.)

**DOI:** 10.3390/ijms23115917

**Published:** 2022-05-25

**Authors:** Hong Yue, Haobin Zhang, Ning Su, Xuming Sun, Qi Zhao, Song Weining, Xiaojun Nie, Wenjie Yue

**Affiliations:** College of Agronomy, State Key Laboratory of Crop Stress Biology in Arid Areas, Northwest A&F University, 3 Taicheng Road, Yangling, Xianyang 712100, China; yuehong@nwsuaf.edu.cn (H.Y.); zhb626@nwafu.edu.cn (H.Z.); sunnying@nwafu.edu.cn (N.S.); sunxm@nwafu.edu.cn (X.S.); zhaoqi1204@nwafu.edu.cn (Q.Z.); sweining2002@outlook.com (S.W.); small@nwsuaf.edu.cn (X.N.)

**Keywords:** drought training, high-throughput sequencing, transcriptional memory, *miR531*, proline

## Abstract

Drought has gradually become one of the most severe abiotic stresses on plants. Plants that experience stress training can exhibit enhanced stress tolerance. According to MicroRNA (miRNA) sequencing data, this study identified 195 candidate drought memory-related miRNAs in wheat, and targets of 64 (32.8%) candidate miRNAs were validated by degradome sequencing. Several drought memory-related miRNAs such as *tae-miR9676-5p*, *tae-MIR9676-p3_1ss21GA*, *tae-miR171a*, *tae-miR531_L-2*, *tae-miR408_L-1*, *PC-3p-5049_3565*, *tae-miR396c-5p*, *tae-miR9778*, *tae-miR164a-5p*, and *tae-miR9662a-3p* were validated as having a strong response to drought memory by regulating the expression of their target genes. In addition, overexpression of drought memory-related miRNA, *tae-miR531_L-2*, can remarkably improve the drought tolerance of transgenic *Arabidopsis* *thaliana*. Drought memory can regulate plant cellular signal transduction, plant biosynthetic processes, and other biological processes to cope with drought via transcriptional memory. In addition, drought memory-related miRNAs can promote starch and sucrose catabolism and soluble sugar accumulation and regulate proline homeostasis to improve plant drought resistance. Our results could contribute to an understanding of drought memory in wheat seedlings and may provide a new strategy for drought-resistant breeding.

## 1. Introduction

Drought is one of the most critical threats to agriculture because of the increased water resource scarcity [1]. As the global climate changes, the frequency, severity, and extent of drought tend to further aggravate agriculture [2]. Wheat (*Triticum aestivum* L.) is the most widely cultivated crop, grown on 210 million hectares, and provides 30% of the food consumed worldwide [3]. It is estimated that by 2050, global wheat yields must increase by 60% to meet the needs of a rapidly growing population [4]. However, wheat production has been falling significantly due to various abiotic stresses, especially drought [5].

Because the environment is constantly changing, plants may experience recurrent environmental stresses throughout their entire life cycle [6]. The molecular mechanisms of plant response to similar abiotic stresses are different from its first encounter [7]. It was reported that many plants could enhance their tolerance to the same pressure [8,9,10]. These quicker and more aggressive responses include structural, genetic, and biochemical modifications that may be defined as stress memory [8,11]. A growing body of research has focused on stress memory in plants [12]. In *Arabidopsis thaliana* (L.) Heynh, stress memory develops through a phenomenon similar to genomic imprinting when exposed to stress [13], and the epigenetic regulation of the proline biosynthetic genes in developing stress memory represents a new research area [14]. Stress memory has a genetic effect on plants. The cold memory was inherited maternally but not paternally in the zygote and early embryos [15]. The H3K27me3 demethylase-HSFA2 regulatory loop can orchestrate transgenerational thermomemory in *Arabidopsis thaliana* [16]. Manipulating MSH1 gene expression allows offspring to inherit the ‘stress memory’ from previous generations, giving them the potential for more robust and more productive growth [17].

Some preliminary studies have been conducted on drought memory. It was reported that drought memory exists in grass over an entire vegetation period, and even in sprouts after harvest [10]. A stalled RNA polymerase II is involved in transcriptional memory during recurring dehydration stresses in *Arabidopsis thaliana*, and four new dehydration stress memory genes display a transcriptional memory response [18,19]. Plant hormones are essential to drought memory. For example, abscisic acid (ABA) could induce the RD29B gene to regulate drought memory [20]. In addition, many studies of drought memory have been conducted on crops. The long non-coding RNA (lncRNA) and DNA methylation, and endogenous phytohormones (especially ABA) may function as memory factors and participate in short-term drought memory in rice [21]. Interestingly, wheat seed osmopriming resulted in long-lasting stress memory and stabilized plant growth and productivity [22]. Furthermore, memory genes related to the plant dehydration response systems may be conserved [23].

MicroRNAs (miRNAs) can regulate plant growth and development through post-transcriptional or translational gene regulation [24]. Studies have shown that miRNAs are involved in various plant regulatory pathways, including epigenetic control of transposable elements, metabolism, and hormone responses, to respond to abiotic stresses [25]. For a long time, numerous studies on the mechanism of drought resistance in wheat have focused chiefly on identifying drought-related protein-coding genes and mining their biological functions [26]. The role of miRNAs in wheat response to drought stress has attracted significant attention. *TaMIR1119* could regulate wheat drought tolerance by transcriptionally regulating the target genes related to osmolyte accumulation, photosynthetic function, and cellular reactive oxygen species (ROS) homeostasis [27]. By establishing miRNA expression profiles in drought-resistant wild emmer wheat, 13 drought response miRNAs were identified [28]. In addition, *miR1435*, *miR5024*, and *miR7714* were up-regulated in the root of durum wheat under drought stress [29]. Some studies also reported that highly dynamic molecules, including miRNA, might contribute to plant stress memory [30]. Many studies have been performed to identify drought response miRNAs in different plant species [31]. Still, little is known regarding the molecular mechanism and biological effects of drought memory response miRNAs in wheat.

Starting from the crop wild relatives (CWR), including those belonging to the genus Aegilops, from which humans began to select the different species of wheat cultivated today [32,33,34], this study identified drought memory response miRNAs and their target genes in wheat by small RNA and degradome sequencing and further analyzed the biological functions of candidate miRNAs. Our results provide insight into wheat drought memory and its possible molecular mechanisms and would be of great significance in breeding new drought-resistant wheat varieties.

## 2. Results

### 2.1. The Influence of Drought Memory to Plant Drought Resistance

**Figure 1:**
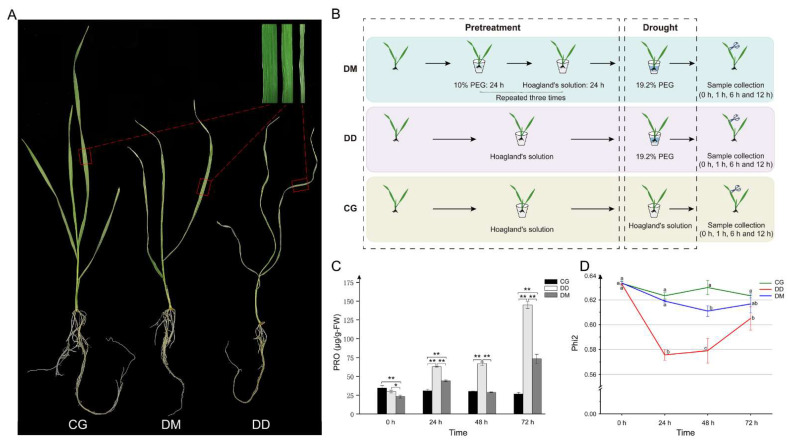
Experimental design and physiological index measurement. (**A**) Drought resistance detection of seedlings in three groups. Trileaf stage wheat seedlings in DD and DM groups were treated with half-strength Hoagland’s liquid medium containing 19.2% PEG for six days, and seedlings grown in normal conditions were used as control. (**B**) Trileaf stage seedlings were divided into three groups, including the control (CG), direct drought (DD), and drought memory (DM). The DM group was pre-treated with 10% (M/V) PEG. Seedlings of the DD and DM groups were then treated with 19.2% PEG, while CG was grown under normal conditions. Whole seedlings of three groups were collected at 0, 1, 6, and 12 h with three biological repetitions and immediately frozen in liquid nitrogen. (**C**) Proline content comparison amongst the three groups after treatment (19.2% (M/V) PEG) for 0, 24, 48, and 72 h. * *p* < 0.05; ** *p* < 0.01. (**D**) Comparison of the efficiency of photosystem II (Phi2) amongst the three groups after treatment (19.2% (M/V) PEG) for 0, 24, 48, and 72 h. Error bars represent the SD. Points on the polyline followed by different letters are statistically different according to the analysis of variance followed by Duncan’s Multiple Range Test (Comparison of different treatments at the same time).

The drought resistance of wheat seedlings that experienced drought training previously could be improved (Figure 1A). The proline content and Phi2 of the samples following three treatments at 0, 24, 48, and 72 h were measured (Figure 1C,D). The proline accumulation in the DM group was significantly higher than CG after 24 h and 72 h drought treatment and lower than in the DD group at all time points. In addition, the photosynthetic efficiency of the DM group was significantly higher than the DD group. This observation means that drought memory pre-treated wheat under severe drought conditions is less damaged than non-drought-trained wheat. Therefore, drought memory wheat needs less proline to maintain cellular homeostasis and preserve higher photosynthetic efficiency than non-drought pre-treated wheat.

### 2.2. High-Throughput Sequencing of miRNAs

Raw reads were first stripped of the 3′ connector sequence and the low-quality reads (Table S1). After removing the PCR duplications and data filtering, 68.9 million unique raw reads were obtained. After filtering by comparing with the mRNA, Rfam, and Repbase databases, 30.0 million unique high-quality valid reads were retained for further analysis (Table S2). Twenty-four nucleotides miRNA represented the highest abundance and accounted for 49.4% of all detected miRNAs. Different length miRNAs belonging to the same miRNA family may have diverse functions [35]. The miRNA length proportion differed amongst the three groups and four time points, suggesting that varying length miRNAs may play diverse roles in drought tolerance and drought memory (Figure S1A).

### 2.3. Identification of Conserved, Known and Novel miRNAs

Unique sequences of 18–25 nucleotides (nt) were mapped to wheat precursors of miRBase 22.0 using a BLAST search to identify miRNAs (Table 1, Figure S1D). Our results identified 644 known miRNAs (gp1a) and 837 conserved miRNAs (gp1b, gp2a, gp2b, and gp3). Amongst these, 141 known miRNAs and 222 conserved miRNAs exist in all libraries (Table S3). After eliminating low expressed miRNAs, 146 novel miRNAs were predicted, of which 61 miRNAs were common to all of the 33 miRNA libraries (Table S4). Except for miRNAs *PC-3p-1639_10290* and *PC-5p-576_26392*, which were expressed at high levels, most of the novel miRNAs exhibited moderate expression level categories. The base distribution for each position of known, conserved, and novel miRNAs was analyzed to investigate base preference. We also found that the first base preference is largely relevant to the length of the miRNA (Figure S1B,C,E,F).

### 2.4. Identification of Drought Memory-Related miRNAs

After drought pretreatment, a total of 198 significantly (*p*-value < 0.05) differentially expressed miRNAs were found (DM_0 h vs. CG_0 h). Of these, 75 miRNAs were down-regulated, and the remainder were up-regulated expressed. Furthermore, 18 differentially expressed miRNAs exhibited a high expression level (Figure S2A). Simultaneously, 228 direct drought response-related miRNAs (union set of DD_1 h vs. CG_1 h, DD_6 h vs. CG_6 h and DD_12 h vs. CG_12 h) were identified (Figure S3).

**Figure 2:**
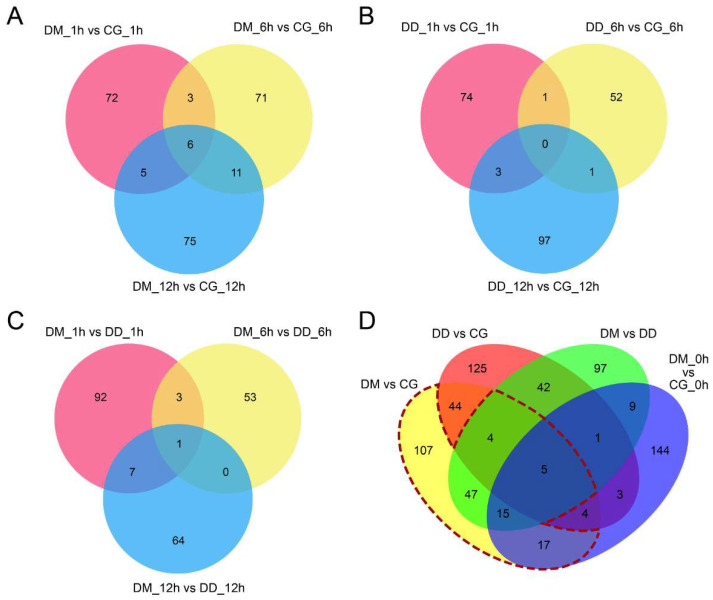
Identification of drought memory-related miRNAs. (**A**–**C**) Venn diagrams of significantly different expressed miRNAs amongst CG, DD, and DM groups (**D**) Identification of drought memory-related miRNAs according to miRNA sequence data. (DM vs. CG) vs. (DD vs. CG) revealed 186 drought memory-specific miRNAs and 57 shared miRNAs. Fifty-seven common miRNAs were searched in the DM vs. DD miRNA set, and nine miRNAs were found to be significantly differentially expressed between DM and DD groups. Finally, we obtained 195 candidate drought memory-related miRNAs (inside the dotted red line).

Direct drought resistance and drought memory are two kinds of drought response mechanisms, and there are both similarities and differences between them. After pretreatment with a periodic repetition of low-level drought (10% PEG), wheat may respond to drought more quickly or aggressively through structural, genetic, and biochemical modifications. When the pre-treated wheat seedlings suffered a higher level of drought stress (19.2% PEG), 243 miRNAs (union set of DM_1 h vs. CG_1 h, DM_6 h vs. CG_6 h, and DM_12 h vs. CG_12 h) were significantly differentially expressed, and they included 128 known and conserved miRNAs and 115 novel miRNAs (Figure 2A and Figure S2B–D). However, when drought pretreatment plants encounter a persistent severe drought, miRNAs related to drought memory and direct drought response are both expressed. Consequently, 57 miRNAs that were common to direct drought and drought memory were removed from the 243 drought memory-related miRNAs. Furthermore, nine miRNAs, included in 57 shared miRNAs but significantly differentially expressed between DM and DD groups, were also considered drought memory-related miRNAs. Finally, 195 miRNAs, including 186 drought memory-specific and nine significantly differentially expressed shared miRNAs, were identified as candidate drought memory-related miRNAs (Figure 2A–D).

### 2.5. Validation of Target Genes of Drought Memory-Related miRNAs in Wheat Using Degradome Sequencing

Degradome sequencing analysis was conducted to narrow down the prediction range of miRNA-target pairs and avoid false positives caused by the prediction software. The unique transcript-mapped reads were obtained from the CG, DD, and DM libraries, respectively (Table S5). Cleavage classifies ‘target plots’ (T-plots) peaks into four categories (0–4), with peaks of categories 0–3 having >1 read per peak, and examples of T-plot for the targets of wheat miRNAs are shown in Figure S4.

Target genes of 195 candidate drought memory-related miRNAs were also validated in the degradome results, with categories <3 as a threshold. Sixty-four (32.8%) candidate drought memory-related miRNAs with 445 target transcripts were detected (Table S6). Among them, 12 miRNAs were expressed at a high level, 37 at a middle level, and 15 at a low level. It is worth mentioning that eight novel miRNAs were also detected, and the pre-miRNA stem-loop secondary structure of these putative drought memory-related miRNAs is shown in Figure S5A. All the mature sequences of the novel wheat miRNAs are observed in the stem regions of the stem-loop structures. Furthermore, 19 miRNAs significantly differentially expressed in pretreatment drought (DM_0 h vs. CG_0 h) can also be detected in these 64 validated drought memory-related miRNAs (Figure S5B).

Enrichment analysis revealed that regulation of transcription, DNA-templated (GO:0006355), DNA binding (GO:0003677), and nucleus (GO:0005634) were the top three GO terms that were significantly enriched in the biological process, molecular function, and cellular component, respectively. Targets of drought memory-related miRNAs were annotated to respond to the hormone, especially the auxin-activated signaling pathway (GO:0009734). Furthermore, drought memory-related transcripts were annotated to ion transport GO terms, such as calcium ion transport (GO:0006816), cadmium ion transport (GO:0015691), iron ion transport (GO:0006811), and response to zinc ion (GO:0010043). Furthermore, the GO terms of histone modification (GO:0016570), seed development (GO:0048316), and leaf development (GO:0048366) were also found in our results (Figure S5C). Phenylalanine metabolism (ko00360), pyruvate metabolism (ko00620), phenylpropanoid biosynthesis (ko00940), and fructose and mannose metabolism (ko00051) are four significantly enriched KEGG pathways. Target transcript of the drought memory-related miRNA *tae-miR164a-5p* annotated to the environmental information processing-mTOR signaling pathway (ko04150) and targets of the *tae-MIR1862d-p3_1ss20TC* are taking part in the glutathione metabolism pathway (ko00480). In addition, the glucose metabolism and amino acid metabolism pathways were also enriched in our results (Figure S5D).

### 2.6. Validation of Drought Memory-Related miRNAs and Their Target Genes by RT-qPCR

**Figure 3:**
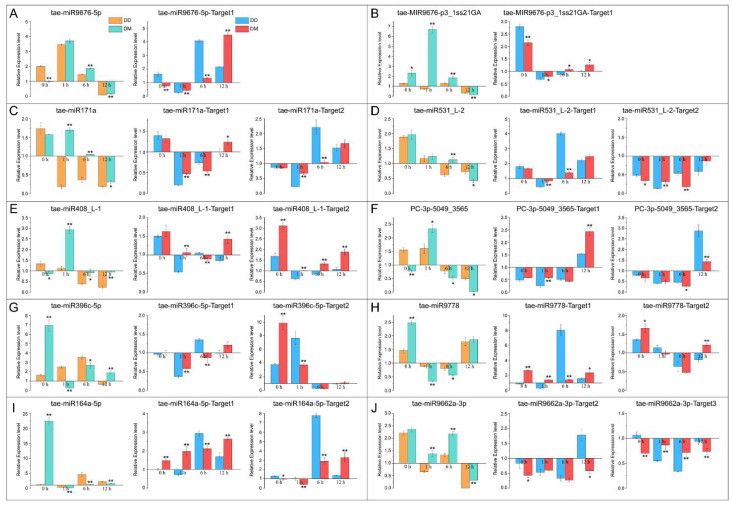
Validation of 10 drought memory-related miRNAs and their target genes by RT-qPCR. (**A**–**J**) The relative expression level of ten candidate drought memory-related miRNAs and their target genes. The relative expression level of each miRNA and their target genes was compared between DD and DM groups at 0, 1, 6, and 12 h, with the CG as control. The expression levels of the miRNAs and target genes were normalized using U6 (GenBank: X63066.1) and Elongation Factor 1-Alpha genes, respectively. All reactions were performed in triplicate for each sample, and expression levels were calculated according to the 2^−ΔΔCT^ method. Error bars represent the SD, * *p* < 0.05; ** *p* < 0.01.

Ten drought memory-related miRNAs, including nine known and conserved miRNAs and one novel miRNA, and their target genes were selected for validation by RT-qPCR based on the abundance and predicted functions (Figure 3). 

Our results indicated that the expression patterns of five drought memory-related miRNAs, including *tae-miR9676-5p*, *tae-miR408_L-1*, *PC-3p-5049_3565*, *tae-miR9778*, and *tae-miR164a-5p*, were similar between the DD and DM groups; however, their expression levels were significantly different between the two groups, and the remaining five miRNAs showed opposite expression patterns between groups DD and DM. Furthermore, the expression patterns of almost all miRNA-target pairs were contrasting in the DM group, except for *tae-miR396c-5p* and *tae-miR9778*. These results confirm the reliability of our sequencing results and indicate that selected candidate miRNAs and their target genes may play crucial roles in drought memory. Moreover, the expression level of drought memory-related miRNAs revealed that drought memory-induced drought response and direct drought response were two different but interrelated processes.

### 2.7. Overexpression of the tae-miR531_L-2 Precursor in Arabidopsis thaliana

The precursor of *tae-miR531_L-2* was overexpressed in *Arabidopsis thaliana* to evaluate the roles of drought memory-related miRNAs in plant drought resistance. 

**Figure 4:**
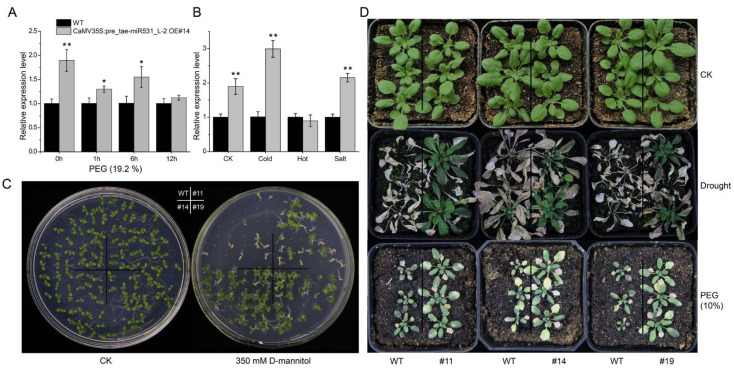
Overexpression of the *tae-miR531_L-2* precursor in *Arabidopsis thaliana*. (**A**,**B**) The expression level of *tae-miR531_L-2* in T_3_ transgenic line (*tae-miR531_L-2*) and wild type (WT) seedlings under drought (20% PEG, 0 h, 1 h, 6 h and 12 h), cold (4 °C, 6 h), heat (37 °C, 6 h), and salt (300 mM NaCl, 6 h) stresses. Error bars represent the SD, * *p* < 0.05; ** *p* < 0.01. (**C**) Germination rate experiment of transgenic lines and WT. Seeds of WT and transgenic lines were germinated on half-strength Murashige and Skoog (MS) agar plates containing 0 mM and 350 mM D-mannitol for two weeks. (**D**) Drought resistance detection in the seedling stage. Two-week-old soil-grown seedlings were air-dried and then rehydrated to detect extreme drought resistance. The seven-day-old soil-grown seedlings were watered with 10% PEG solution for two weeks for drought resistance detection under prolonged mild drought.

Our results found that *tae-miR531_L-2* was significantly overexpressed in transgenic *Arabidopsis thaliana*, and the expression level was significantly higher than WT under drought stress. In addition, *tae-miR531_L-2* can also strongly respond to cold and salt stresses, but there was no significant response to heat stress (Figure 4A,B). Our phenotypic experiment demonstrated that the overexpression of drought memory-related miRNA *tae-miR531_L-2* could significantly improve the drought resistance of *Arabidopsis thaliana* in many ways. It can also considerably enhance the seed germination and seedling survival rates under drought stress (Figure 4C). On the other hand, the overexpression of *tae-miR531_L-2* could not only remarkably improve the survival rate of *Arabidopsis thaliana* under severe soil drought but also considerably improve the seedling biomass and seedling growth rate under prolonged mild drought conditions (Figure 4D).

## 3. Discussion

### 3.1. Identification of Drought Memory-Related miRNAs and Their Target Genes in Wheat

Drought memory is a complex biological process regulated by the expression of many genes. Previous studies have reported that *miR156*, *miR391*, and *miR831* are heat stress memory-related miRNAs in *Arabidopsis thaliana*, and *miR398* and *miR408* were associated with drought memory in coffee plants [36,37]. Our results suggest that some of the above stress memory-related miRNAs are equally important in drought memory-induced drought resistance. In this study, the *tae-miR408_L-1* may be involved in drought memory by regulating the expression of genes associated with the electron transport chain and metal ion binding. Some miRNAs involved in direct drought may also play a role in drought memory. Accumulation studies have shown that many miRNAs, including *miRNA408*, *171*, *164*, and *396*, play essential roles in the drought stress resistance of plants [38,39]. Our results found that the drought memory-related miRNA *tae-miR408_L-1* is indispensable in drought memory and direct drought response. In addition, the drought memory-related miRNA *tae-miR164a-5p* could target MAP kinase activity (GO:0004707) and mTOR signaling pathway (ko04150) related transcripts, which might improve the drought tolerance of wheat [40,41]. Target transcript of *tae-miR396c-5p* was associated not only with abscisic acid (GO:0009737) and mannitol (GO:0010555) but were also positively regulated in response to water deprivation (GO:1902584). ABA is a crucial drought memory-related plant hormone of rice [21]. The *miR171* in rice might improve salt tolerance by modulating physiological changes, stomatal development, ABA-dependent pathways, and expression of stress-related genes [42]. Our results showed that the drought memory-related miRNA, *tae-miR171a*, could respond to both direct drought and drought memory, and the expression patterns were different from each other (Figure 3C).

Our study also identified some new drought memory-related miRNAs. A novel drought memory-related miRNA, *PC-3p-5049_3565*, was detected, and it targeted fructose bisphosphate aldolase (FBA). It has been reported that FBA is a salt stress-responsive marker protein in chloroplasts that catalyzes the cleavage of fructose-1-6-bisphosphate into D-glyceraldehyde-3-phosphate, thus producing dihydroxyacetone phosphate and adenosine triphosphate [43]. Moreover, FBA may be one of the target proteins responsible for Al stress in wheat roots [44]. The function of FBA in drought stress memory in wheat deserves to be studied further.

### 3.2. miRNA-Gene-GO Association Analysis of Drought Memory-Related miRNAs

**Figure 5:**
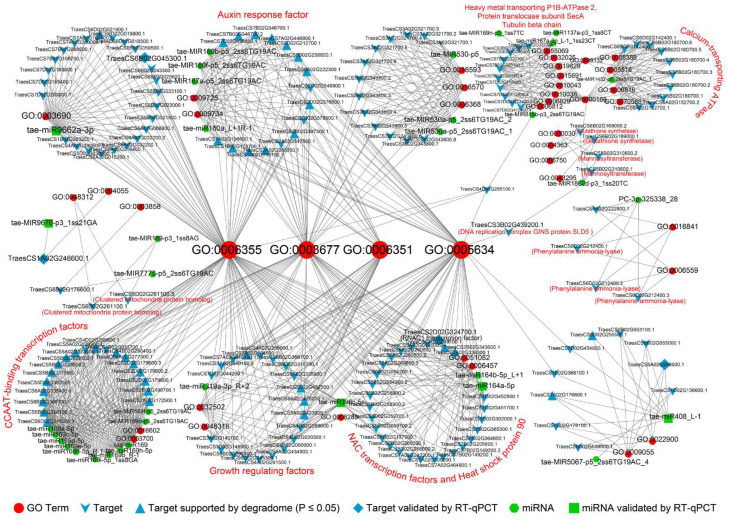
The miRNA-gene-GO association analysis of drought memory-related miRNAs. Candidate drought memory-related miRNAs, their target transcripts, and annotated GO terms were used to investigate the relationship between them. The miRNAs are denoted in green, target transcripts are denoted in cyan, and the GO terms are red. The annotated functions of target transcripts are labeled in red.

Enrichment analysis was conducted amongst target transcripts of 64 drought memory-related miRNAs and used 42 significantly (*p* < 0.05) enriched GO terms for association analysis (Figure 5). The results revealed that drought memory displayed a transcriptional stress memory (GO:0003677, GO:0006355, GO:0006351, and GO:0005634). Transcriptional stress memory can respond to drought by regulating other specific functions. The present study found that drought memory wheat may respond to severe drought by regulating plant cellular signal transduction. Firstly, drought memory might regulate the multiple signaling pathways by drought memory-related miRNAs (*tae-miR160a_L+1R-1*, *tae-MIR167a-p5_2ss6TG19AC*, *tae-MIR160f-p5_2ss6TG19AC*, and *tae-MIR160b-p5_2ss6TG19AC*) that target transcripts that significantly enriched the auxin-activated signaling pathway (GO:0009734). What is more, drought memory-related miRNA *tae-MIR1432-p5_2ss6TG19AC_1* could target calcium-transporting ATPase genes. The exogenous calcium was reported to modify long-term memory and further control regeneration in plant cells [45], and the regulation of the calcium-sensing receptor in both stomatal movement and photosynthetic electron transport is crucial for water use efficiency and drought tolerance in *Arabidopsis thaliana* [46]. 

Drought memory may also regulate plant biosynthetic processes through transcriptional memory. Drought memory-related miRNA *tae-MIR1862d-p3_1ss20TC* could target glutathione biosynthetic transcripts. Glutathione and its related enzymes have great significance in the response of plants to drought [47]. In addition, miR167 family members, *tae-miR167a-3p_L-1_1ss23CT* and other drought memory-associated miRNAs, are also involved in drought response through the CCAAT-binding factor complex, NAC transcription factors and heat shock protein 90, protein modification, electron transport, energy metabolism, amino acid metabolism, plant growth and development, and P1B-ATPase 2 for heavy metal transport.

### 3.3. Proposed miRNA-Dependent Regulatory Pathways That Participate in Drought Memory

**Figure 6:**
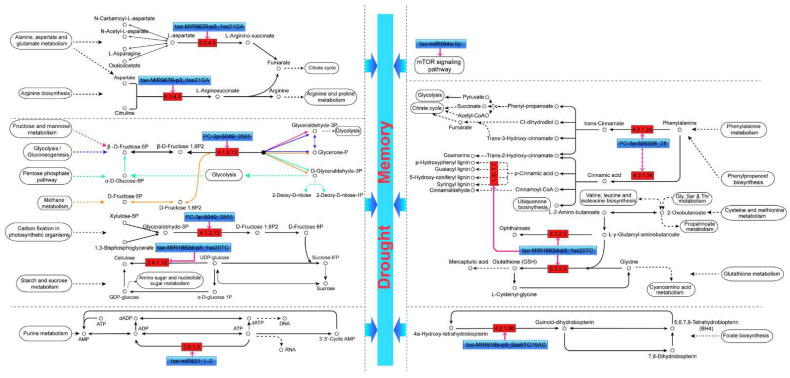
A proposed regulatory mechanism involving the differentially expressed miRNAs and their target genes in wheat drought memory. Targets of eight miRNAs were enriched to fifteen pathways that might be crucial to drought memory.

Drought memory is a complex process that results from the synergistic effects of a series of regulatory pathways, including biosynthesis and metabolism of drought-resistant substances and signal transduction. This study found several pathways that may be important for drought memory (Figure 6). A novel drought memory-related miRNA *PC-3p-5049_3565* targeted two transcripts involved in the transition between glyceraldehyde triphosphate and D-fructose 1,6 diphosphate. Target transcripts of *tae-MIR1862d-p3_1ss20TC* are involved in the starch and sucrose metabolism (ko00500) pathway and are linked to carbon fixation in the photosynthetic organisms pathway via D-fructose-6-phosphate. In addition, *PC-3p-325338_28* and *tae-MIR1862d-p3_1ss20TC* participate in the phenylalanine metabolism (ko00360) and phenylpropanoid biosynthesis (ko00940) pathways. On the one hand, phenylalanine, tyrosine, and tryptophan metabolisms can synthesize proteins, other secondary metabolites, and plant hormones to promote plant metabolism [48]. On the other hand, the down-regulate expression of genes in these pathways could increase carbohydrate metabolism by glycolysis and produce more energy in response to drought [49]. Otherwise, the target transcript of *tae-MIR1862d-p3_1ss20TC* is also involved in glutathione, cysteine, and methionine metabolism pathways to respond to drought memory.

What is more, target transcripts of the *tae-MIR9676-p3_1ss21GA* (*TraesCS1A02G246600.1*) participate in two closely related pathways, including alanine, aspartate, and glutamate metabolism (ko00250) and arginine biosynthesis (ko00220). As the arginine biosynthesis pathway product, arginine is further involved in the arginine and proline metabolism pathways. The proline accumulation under osmotic stress was accompanied by increased soluble sugar concentration [50,51]. Sucrose could inhibit ABA-induced proline accumulation, and ABI4 is involved in ABA and sugar signaling [52]. Our results revealed that proline accumulation in the DM group was significantly slower than that of the DD group (Figure 1C). Therefore, we propose a drought memory-induced drought response model in plants: drought memory-related miRNAs can improve plant drought resistance by promoting starch and sucrose catabolism, soluble sugar accumulation, and regulating proline homeostasis.

## 4. Materials and Methods

### 4.1. Plant Materials and Treatments

‘Chinese Spring’ is a local wheat variety from China that is sensitive to drought. Seeds of the ‘Chinese Spring’ wheat, preserved in our laboratory, were selected as research material for this study. It was germinated on moist filter paper in Petri dishes and then transferred to a half-strength Hoagland’s liquid medium [53]. Seedlings were grown in a growth chamber under controlled conditions (23 ± 1 °C, 16 h light/8 h dark cycle). Trileaf stage seedlings were divided into three groups: control group (CG), direct drought (DD), and drought memory (DM) groups. The DM group was pre-treated with 10% (M/V) PEG for 24 h in Hoagland’s nutrient solution. They were then allowed to grow in Hoagland’s nutrient solution for 24 h, and this treatment was repeated three times [23,54]. At the same time, the CG and DD groups were cultured in Hoagland’s nutrient solution. Subsequently, all seedlings from the DD and DM groups were treated with 19.2% (M/V) PEG, whereas the CG group was grown under normal conditions. Whole seedlings of 33 samples from the three groups at drought treated 0, 1, 6, and 12 h (with three biological repetitions) were collected and immediately frozen in liquid nitrogen for the subsequent test (Figure 1B). Furthermore, the leaves of the three groups (CG, DD, and DM) after treatment (19.2% (M/V) PEG) for 0, 24, 48, and 72 h were collected to measure proline content using a proline assay kit (Nanjing Jiancheng Bioengineering Institute, Nanjing, China) with three biological repetitions, and the photosystem II (Phi2) was measured at 0 to 72 h intervals on leaves using a Photosynq device [55].

### 4.2. Small RNA and Degradome Library Construction and Sequencing

Total RNA was isolated and purified using the TRIzol reagent (Invitrogen, Carlsbad, CA, USA) following the manufacturer’s instructions. The RNA amount and purity of each sample were quantified using a Nano Drop ND-1000 instrument (Nano Drop, Wilmington, DE, USA). Formaldehyde denatured gel electrophoresis was used to assess RNA integrity. UMI labeling technology was then used to construct miRNA libraries to eliminate the effects of polymerase chain reaction (PCR) duplications according to the TruSeq Small RNA Sample Preparation Guide [56]. Finally, the library was sequenced on an IlluminaHiSeq 2500 device with PE150 mode at the LC-BIO (Hangzhou, China).

Every 12 samples representing the four time points (0, 1, 6, and 12 h) in the same group were pooled to construct a degradome library, and three libraries (CG, DD, and DM) were finally constructed. The degradome cDNA library was prepared following the method previously described by Axtell, with some modifications [57]. A 3′-adapter random primer was used to make the first strand of cDNA from mRNA, and size selection was performed using AMPure XP beads. Following the manufacturer’s recommended protocol, the cDNA libraries were 50 base pairs (bp) single-end sequenced on an IlluminaHiSeq 2500 (LC Bio, Hangzhou, China).

### 4.3. Identification of Known and Potential Novel miRNAs

After the Illumina sequencing, the 3′ connector sequence and low-quality data of the raw reads were first stripped, and the 30–80 bp data was retained, then the UMI sequence was extracted to remove PCR duplication and the PCR duplication ratio was calculated. The remaining sequences were subjected to an in-house program, ACGT101-miR_v4.2 (LC Sciences, Houston, TX, USA), to remove adapter dimers, junk, low complexity, common RNA families (rRNA, tRNA, snRNA, snoRNA), and repeats. After this, unique sequences of 18–25 nt were mapped to wheat precursors in miRBase 22.0 (ftp://mirbase.org/pub/mirbase/CURRENT/ (accessed on 1 June 2021)) by BLAST search using Bowtie2 to detect known miRNAs [58]. The unmapped reads were then mapped to multiple databases, including mRNA (ftp://ftp.ensemblgenomes.org/pub/plants/release-41/fasta/triticum_aestivum (accessed on 1 June 2021)), Repbase (http://www.girinst.org/repbase (accessed on 1 June 2021)), Rfam14 (http://rfam.janelia.org (accessed on 1 June 2021)), and genomic DNA (ftp://ftp.ensemblgenomes.org/pub/plants/release-41/fasta/triticum_aestivum (accessed on 1 June 2021)) using Bowtie2. The genome-mapped reads extended on the flanking region and the predicted secondary structure tended to form hairpins that could be considered the novel miRNAs. A modified global normalization was used to correct copy numbers among different samples, and the method corresponded to the previous study [59]. Based on bioinformatics analysis, these miRNA sequences were classified into four groups (gp1–gp4) based on the classification method [60]. gp1a are known miRNAs that have been reported in wheat; gp1b, gp2, and gp3 represent conserved miRNAs; and gp4 contains novel miRNAs.

### 4.4. Analysis of Differentially Expressed miRNAs

The T-test (http://en.wikipedia.org/wiki/Student’s_t-test (accessed on 1 June 2021)) and ANOVA test (http://en.wikipedia.org/wiki/Oneway_analysis_of_variance (accessed on 1 June 2021)) were chosen for pairwise-group and multi-group comparisons, respectively, to identify differential expression of miRNAs based on normalized deep-sequencing counts. The significance threshold was set at a *p*-value < 0.05 in each test. The expression level of the miRNAs was divided into three levels: high, medium, and low. High expression is the number of reads for the miRNAs that were higher than the average copy of the dataset. The medium expression is the number of reads for the miRNAs that were higher than ten reads and less than the average copy of the dataset, and the low expression was the number of reads reported as less than ten.

### 4.5. Degradome Validation and Annotation of Target Genes of miRNAs

ACGT101-DEG software (LC-bio Sciences, Houston, TX, USA) was used to inspect the targets and evaluate the correctness of the identified target, and the CleaveLand (v3.0) program was used to identify potentially cleaved targets [61]. The degradome reads were then mapped to the wheat transcriptome data. According to five categories (0–4), all targets were categorized according to the T-plot peaks as described in previous studies [62,63]. Furthermore, Gene Ontology (GO) and Kyoto Encyclopedia of Genes and Genomes (KEGG) annotation were performed to predict the functions of target genes of the miRNAs according to the information collected from BioMart in Ensembl (http://plants.ensembl.org/Triticum_aestivum/Info/Index (accessed on 1 June 2021)). A hypergeometric test was then used to find the functions or pathways that were significantly enriched in the selected miRNA-mRNA pairs.

### 4.6. Validation of Differentially Expressed miRNAs and Their Target Genes

Reverse transcription-quantitative real-time PCR (RT-qPCR) was conducted to detect the expression level of drought memory-related miRNAs and their target genes in wheat. The sample collection, total RNA extraction, purification, quantification, and integrity detection methods were consistent with those described above. The first-strand cDNAs of miRNAs were synthesized using an miRNA 1st Strand cDNA Synthesis Kit (by stem-loop) (MR101-01, Vazyme, Nanjing, China) with the mixed reference gene reverse transcription (RT) primers and miRNA-specific stem-loop RT-primers. In addition, the first-strand cDNAs were also synthesized using Oligo dT (18T) primer and random 6 mers primer with the Evo M-MLV Mix Kit with gDNA Clean for qPCR (AG11728, Accurate Biotechnology (Hunan) Co., Ltd., Hunan, China). The RT-qPCR analysis was conducted using the QuantStudio™ 7 Flex System (Thermo Fisher Scientific, Waltham, MA, USA) with SYBR^®^ Green Premix Pro Taq HS qPCR Kit (Rox Plus) (AG11718, Accurate Biotechnology (Hunan) Co., Ltd., Hunan, China). The expression levels of the miRNAs and target genes were normalized using U6 (GenBank: X63066.1) and Elongation Factor 1-Alpha genes, respectively. All reactions were performed in triplicate for each sample. The primers used in this study are listed in Table S7, and expression levels were calculated according to the 2^−ΔΔCT^ method [64].

### 4.7. Overexpression of tae-miR531_L-2 in Arabidopsis thaliana

The precursor DNA sequence (70 bp) of the *tae-miR531_L-2* was obtained from the miRNA sequence result, the precursor sequence contained 200 bp upstream and downstream, and the stem-loop sequence was cloned from the wheat genome with the primers F: 5′-GTCAACGTCAGTACCTCATGC-3′ and R: 5′-AACTGCAGCGGAGCATCAACG-3′. The cloned sequence was validated with Sanger sequencing and then inserted into the plant binary expression vector pBI121 by *XbaI* and *KpnI* restriction sites to construct an overexpression vector. This construct was introduced into *Agrobacterium tumefaciens* GV3101 and then transformed into *Arabidopsis thaliana* (Columbia) using the floral dip method [65].

All plants were grown in a growth chamber with 16 h light/8 h dark cycles and 22 °C/19 °C day and night temperature. Soil-grown two-week-old T_3_ homozygous transgenic line and wild type (WT) seedlings treated with salt (300 mM NaCl, 6 h), cold (4 °C, 6 h), heat (37 °C, 6 h), and drought (20% PEG, 0 h, 1 h, 6 h and 12 h) were collected for RT-qPCR analysis. The WT seedlings under the same treatment were used as a control to detect the relative expression level of *tae-miR531_L-2* in transgenic *Arabidopsis thaliana*. Methods of total RNA extraction, first-strand cDNA synthesis, RT-qPCR, and relative expression level calculation were the same as those described above. For the drought resistance experiment of transgenic lines and WT, (1) seeds were surface-sterilized and germinated on half-strength Murashige and Skoog (MS) agar plates containing 0 mM and 350 mM D-mannitol for two weeks; (2) two-week-old soil-grown seedlings were air-dried and then rehydrated; (3) seven-day-old soil-grown seedlings were watered with 10% PEG solution for two weeks.

## 5. Conclusions

Repeated drought training can improve drought resistance in wheat through miRNA regulatory networks. Drought memory can regulate plant cellular signal transduction, plant biosynthetic processes, and other biological processes to cope with drought via miRNA-regulated transcriptional memory. This study proposes a model of drought memory-induced drought response in plants: drought memory-related miRNAs can improve plant drought resistance by promoting starch and sucrose catabolism and soluble sugar accumulation as well as by regulating proline homeostasis. Furthermore, overexpression of the drought memory-related miRNA *tae-miR531_L-2* in plants significantly improved the drought resistance of transgenic *Arabidopsis thaliana*. The results presented here might provide novel insight into resolving wheat drought tolerance mechanisms and drought-resistant breeding.

## Figures and Tables

**Table 1:** The miRNA sequence data overview.

Sample	UMI Labeled Non-Redundant Data	Known miRNAs	Conserved miRNAs					Novel miRNAs
		gp1a	gp1b	gp2a	gp2b	gp3	total	gp4
CG/DD_0 h	8244816	461	210	330	42	71	653	1431
CG_1 h	9284052	414	217	344	38	65	664	1306
CG_6 h	8631839	495	211	282	39	68	600	1470
CG_12 h	11244731	437	211	356	42	68	677	1294
DD_1 h	8829616	372	212	325	38	54	629	1292
DD_6 h	11247192	469	216	355	41	64	676	1430
DD_12 h	18968451	407	216	374	39	72	701	1419
DM_0 h	10502181	490	220	348	38	64	670	1754
DM_1 h	7799044	348	214	329	36	63	642	1226
DM_6 h	12679727	332	202	306	41	56	605	1333
DM_12 h	13278172	401	211	332	36	59	638	1159

## Data Availability

The raw reads, including small RNA and degradome sequencing, in this study are publicly available at the NCBI Sequence Read Archive (SRA) under accession codes PRJNA799639 and PRJNA799641, respectively.

## Supplementary Materials

The following supporting information can be downloaded at: https://www.mdpi.com/article/10.3390/ijms23115917/s1.
