# Supplementary material for: Integrate Small RNA and Degradome Sequencing to Reveal Drought Memory Response in Wheat (Triticum aestivum L.)
Source: Int J Mol Sci. 2022 May 25;23(11):5917. doi: 10.3390/ijms23115917 (PMC9180835; doi:10.3390/ijms23115917)
Supplement: Supplementary file 1 [file ijms-23-05917-s001.zip › ijms-1728728-supplementary/Supplementary Figures.pdf]

Support Figures

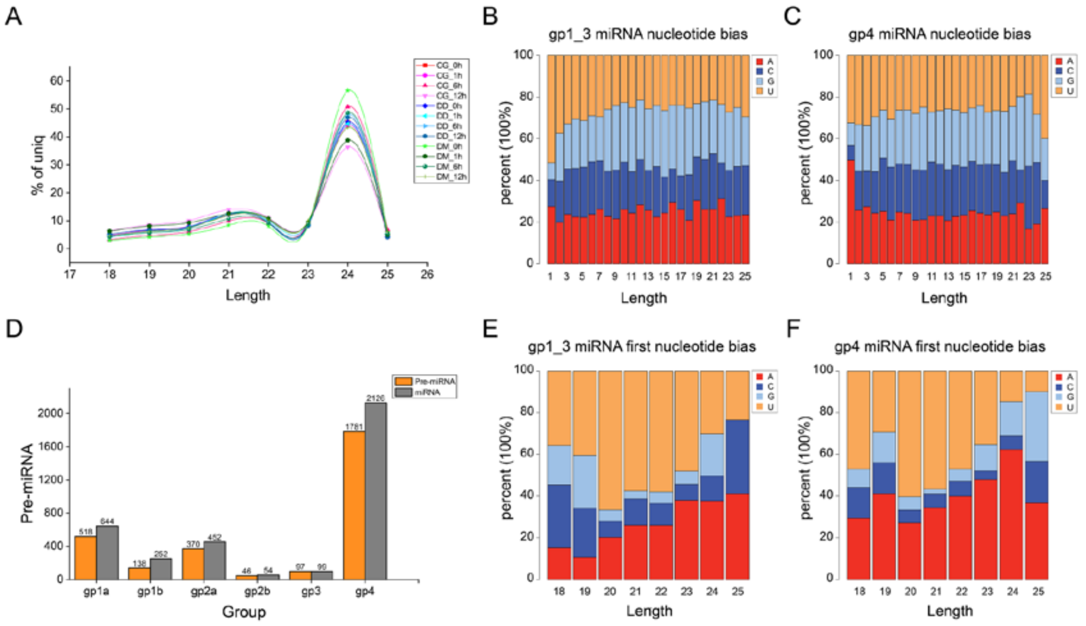

Figure S1. The miRNA sequence data overview. (**A**) The length distribution of the unique valid reads in all of the samples. (**B, C**) The base distribution for each position of known, conserved and novel miRNAs. (**D**) Identification of known, conserved and novel miRNAs. Gp1a are known miRNAs; gp1b, gp2a, gp2b and gp3 are conserved miRNAs; and gp4 represents novel miRNAs. (**E, F**) The first nucleotide bias of known, conserved and novel miRNAs.

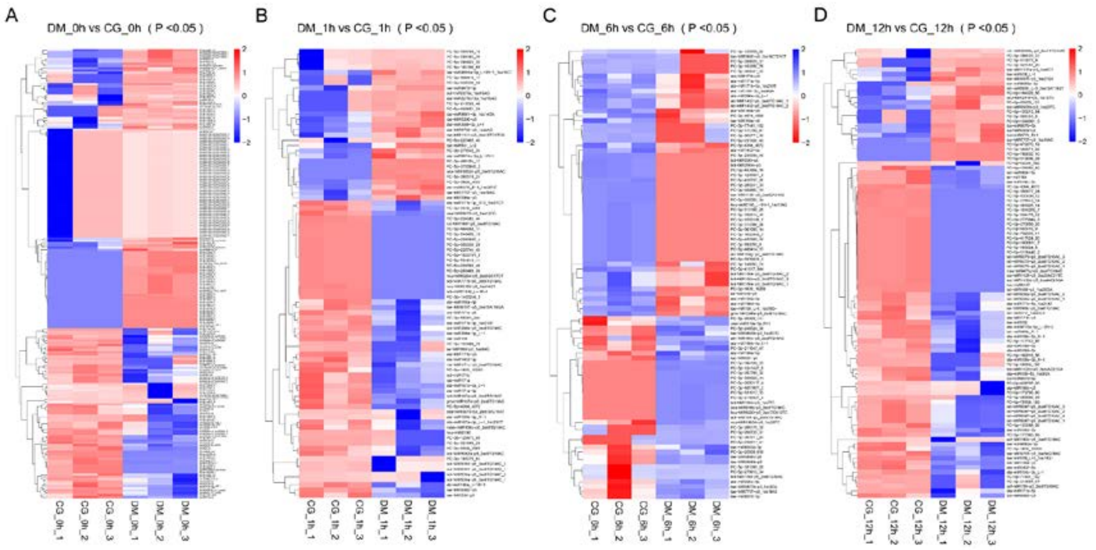

Figure S2. Significantly differentially expressed miRNAs between DM and CG groups. (**A**) The expression patterns of 198 pre-treatment significantly differentially expressed miRNAs (DM_0 h vs CG_0 h). (**B-D**) The expression pattern of 243 significantly ($p < 0.05$) differentially expressed miRNAs in pre-treated wheat seedlings that suffered severe drought. The red, white and blue colors represent the higher to the lower relative abundance of each miRNA.

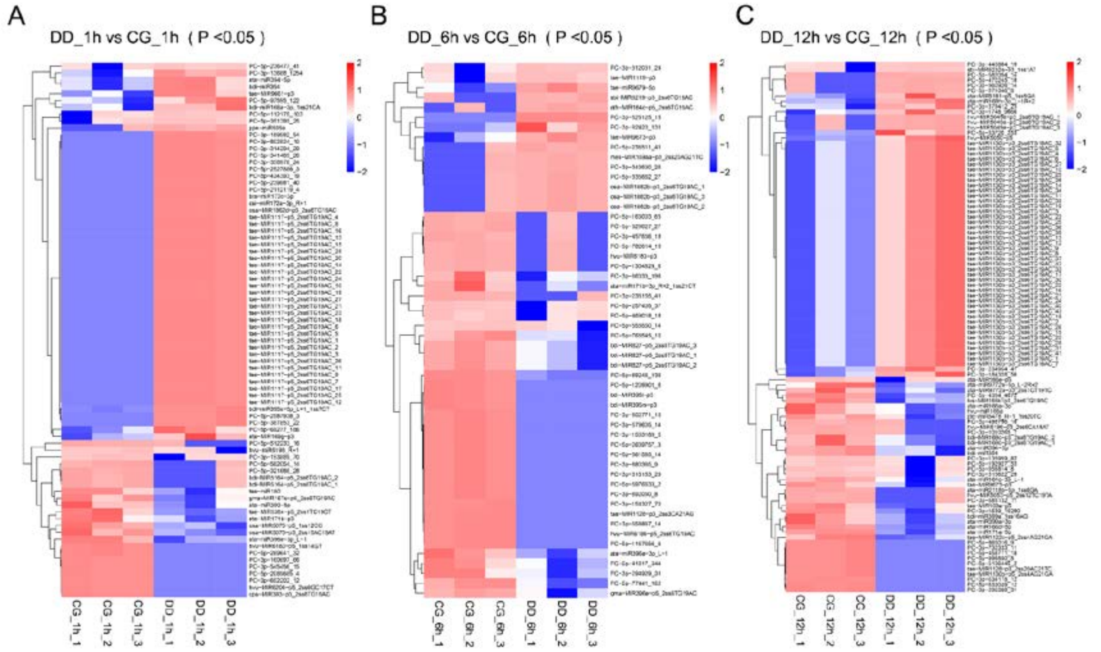

Figure S3. The expression patterns of direct drought-related miRNAs. There are 228 significantly differentially expressed miRNAs between DD and CG treatments (union set of DD_1 h vs. CG_1 h, DD_6 h vs. CG_6 h and DD_12 h vs. CG_12 h). The red, white and blue colors represent the higher to the lower relative abundance of each miRNA.

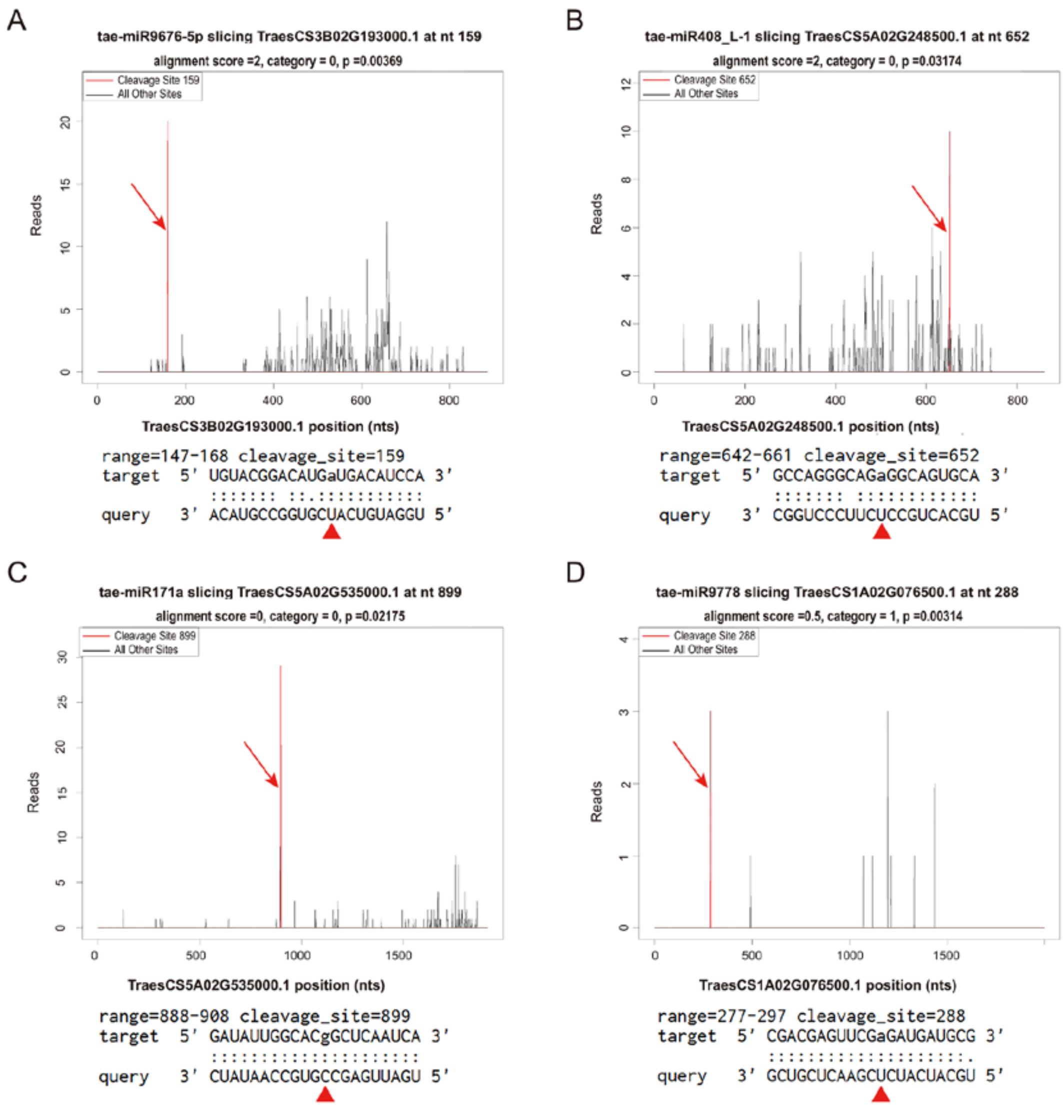

Figure S4. Examples of degradome sequencing verified targets of drought memory-related miRNAs. The T-plots was shown the degradome tags' distribution and the full-length target mRNA sequence. The red line indicates the cleavage site of each transcript and is also shown by an arrow. The red triangle indicates the specific cleavage base site in the target gene sequence.

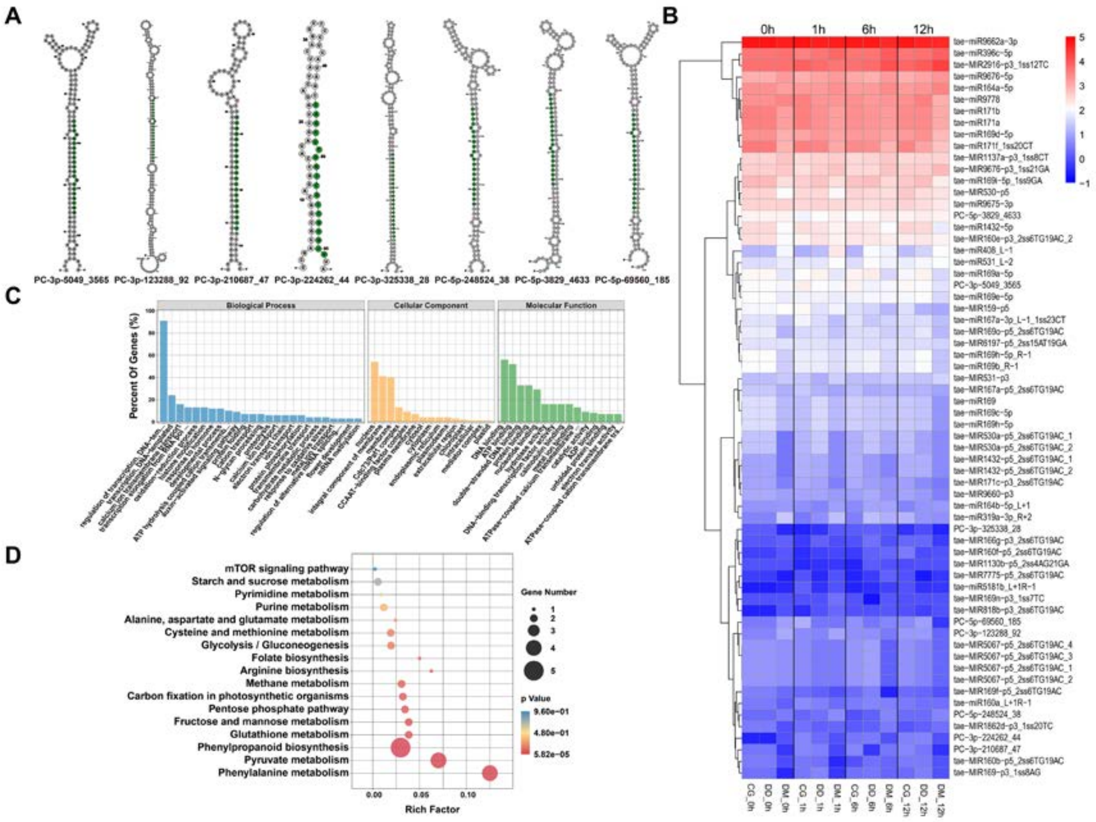

Figure S5. The characteristics of 64 drought memory-related miRNAs. (**A**) The pre-miRNA stem-loop secondary structure of novel drought memory-related miRNAs. (**B**) The expression patterns of 64 drought memory-related miRNAs. (**C-D**) The GO and KEGG enrichment analysis of the candidate drought memory-related targets.
